# Patterns of daily oral HIV PrEP adherence among people who inject drugs in Ukraine: an analysis of biomarkers

**DOI:** 10.1002/jia2.26319

**Published:** 2024-07-19

**Authors:** Olga Morozova, Marina Kornilova, Olena Makarenko, Svitlana Antoniak, Mariia Liulchuk, Olga Varetska, Kostyantyn Dumchev

**Affiliations:** ^1^ Biological Sciences Division Department of Public Health Sciences University of Chicago Chicago Illinois USA; ^2^ International Charitable Foundation “Alliance for Public Health” Kyiv Ukraine; ^3^ Ukrainian Institute on Public Health Policy Kyiv Ukraine; ^4^ Gromashevsky Institute of Epidemiology and Infectious Diseases National Academy of Medical Sciences of Ukraine Kyiv Ukraine

**Keywords:** tenofovir, emtricitabine, tenofovir diphosphate, emtricitabine triphosphate, dried blood spot, biomarkers

## Abstract

**Introduction:**

Daily oral HIV pre‐exposure prophylaxis (PrEP) with tenofovir/emtricitabine (TDF/FTC) is recommended for people who inject drugs (PWID) but coverage is low. The real‐life effectiveness of PrEP among PWID is unknown as previous studies were conducted in controlled settings and mainly relied on self‐report. Analysis of PrEP metabolites—tenofovir diphosphate (TFVdp) and emtricitabine triphosphate (FTCtp)—offers an objective measure of adherence.

**Methods:**

To analyse longitudinal patterns of PrEP adherence among PWID in Ukraine, we used data from a community‐based implementation trial conducted in Kyiv between July 2020 and March 2021 to test the efficacy of SMS reminders to improve adherence. Among 199 enrolled participants, 156 (78.4%) were retained through 6 months. Based on TFVdp/FTCtp levels assessed at 3 and 6 months, we identified groups with various adherence patterns (adherent at ≥2 doses/week, improved, worsened, non‐adherent). Correlates of adherence were analysed using multinomial logistic regression.

**Results:**

Most participants (53.8%, *n* = 84/156) had no detectable metabolites at both assessments; 7.1% (*n* = 11/156) were consistently taking ≥2 doses/week; 1.3% (*n* = 2/156) were consistently taking ≥4 doses/week; 13.5% (*n* = 21/156) exhibited improved and 21.8% (*n* = 34/156) had worsened adherence at 6 compared to 3 months. “White coat compliance” (increased dosing prior to assessment) was common. Consistent adherence was associated with SMS reminders, younger age, employment, lower income, longer injection drug use duration, recent high‐risk injecting (receptive syringe sharing, using pre‐filled syringe, back‐ or front‐loading, container sharing), absence of overdose in the past 6 months, perceived HIV risk through sexual intercourse and higher PrEP self‐efficacy. Alcohol consumption was associated with inconsistent PrEP use. Groups with improved and worsened adherence did not differ.

**Conclusions:**

Daily oral PrEP may not achieve the desired effectiveness among PWID as a standalone intervention, calling for testing of alternative PrEP formulations and innovative integrated risk reduction strategies, especially in the context of HIV epidemics associated with injection drug use in eastern Europe and central Asia and the public health crisis in Ukraine caused by the war with Russia. SMS reminders may be effective among PWID who prioritize PrEP. Our findings offer practical guidance in identifying PWID who are likely to benefit from PrEP and those who need additional support.

## 1 INTRODUCTION

Daily oral pre‐exposure prophylaxis (PrEP) with tenofovir/emtricitabine (TDF/FTC) is an important component of the HIV prevention toolkit, with efficacy demonstrated among all key populations [1, 2]. Despite the recommendations to prescribe PrEP to people who inject drugs (PWID), coverage remains low, services are often unavailable and national policies are lacking [3, 4, 5, 6, 7, 8]. Evidence of real‐life PrEP effectiveness among PWID is limited [3, 4, 5]. Monitoring of PrEP adherence that is key to its effectiveness is challenging as traditional measures are often inaccurate [9, 10, 11]. Recent advances in the development of biomarkers, including unique pharmacokinetic profiles of PrEP metabolites tenofovir diphosphate (TFVdp) and emtricitabine triphosphate (FTCtp), allow objective and more accurate measurements of adherence [11, 12, 13, 14] compared to traditional measures. A combination of the two metabolites reduces misclassification compared to each metabolite alone [15].

After the Bangkok Tenofovir Study, only three studies analysed PrEP adherence among PWID using biomarkers [16, 17, 18]. These studies found low adherence and low validity of self‐reported PrEP compliance, but little is known about the patterns and dynamics of adherence and factors associated with PrEP uptake in this population [3, 6], in particular in settings with HIV epidemics concentrated in PWID communities. In Ukraine—one such setting [19, 20, 21, 22]—PrEP has been available since 2017, and national guidelines recommend daily dosing. However, in 2021, 828 PWID were receiving PrEP corresponding to an estimated 0.3% of HIV‐negative PWID [23]. In this study, we analyse data from the PrEP adherence intervention trial conducted in Kyiv, Ukraine [18], to explore patterns and correlates of PrEP adherence among PWID over time.

## 2 METHODS

### 2.1 Study design

The detailed methodology of the parent study was described previously [18]. An implementation trial among 199 PWID was conducted between July 2020 and March 2021 to test the efficacy of SMS reminders in improving PrEP adherence. Participants were recruited by outreach workers of community harm reduction programmes. Consented participants completed structured surveys and provided blood samples (used for dried blood spot [DBS] and HIV testing) at baseline and at 3‐ and 6‐month visits.

### 2.2 Measurement and interpretation of PrEP metabolites (TFVdp/FTCtp)

Details of the DBS sample collection, storage and analyses have been published [18]. The lower limit of quantification was 100 fmol/punch for both metabolites. We used the TFVdp threshold of ≥700 fmol/punch to indicate ≥4 doses/week on average over the past 2−3 months [9]. The interpretation of TFVdp/FTCtp level combinations in DBS was based on pharmacokinetic modelling [15] and population pharmacokinetic studies [12, 13].

### 2.3 Dynamics of PrEP adherence

**Figure 1:**
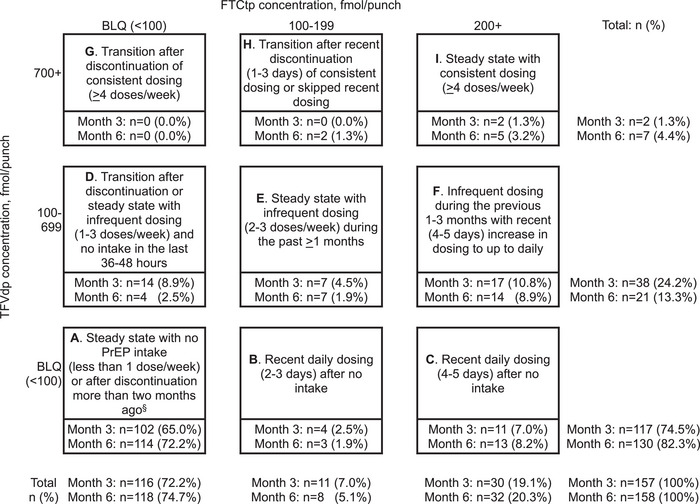
**Observed combinations of TDF/FTC metabolite levels in dried blood spot and their possible interpretations among people who inject drugs in Kyiv, Ukraine during July 2020–March 2021. Cell labels represent pharmacokinetic interpretations of metabolite combinations in the study population and do not capture all possible variation of dosing scenarios**. Abbreviations: BLQ, below the limit of quantification; FTC, emtricitabine; FTCtp, emtricitabine triphosphate; TFV, tenofovir; TFVdp, tenofovir diphosphate. ^§^Recent PrEP intake of a single dose less than 2−8 hours before the blood draw or 3−7 days before the blood draw in the absence of regular intake would result in the FTCtp concentration that falls below the limit of quantification; however, *long‐term* steady state dosing of 1 dose/week is consistent with TFVdp concentration of about 300 fmol/punch, which falls above the limit of quantification for TFVdp.

Based on the changes of TFVdp/FTCtp concentrations between 3‐ and 6‐month assessments (Figure 1), we defined the dynamics of adherence as follows:

*Consistent non‐adherence*: undetectable levels (cell A) at both assessments.

*Worsened adherence*: transition to undetectable levels (cell A) at 6‐month from any other cell at 3‐month, or transition from consistent intake of ≥2 doses/week at 3‐month (cells E/F/H/I) to any other cell at 6‐month.

*Improved adherence*: transition from undetectable levels (cell A) at 3‐month to any other cell at 6‐month, or transition to a consistent intake of ≥2 doses/week at 6‐month (cells E/F/H/I) from any other cell at 3‐month.

*Consistent adherence*: any transitions indicating consistent intake of ≥2 doses/week (cells E/F/H/I).

*Mixed adherence*: all other transitions, for example between cells B and D.

### 2.4 Statistical analysis

Associations between the patterns of PrEP adherence dynamics (consistent non‐adherence, worsened, improved and consistent adherence) as an outcome and potential correlates were analysed in the R computing environment [24] using multinomial logistic regression [25]. Covariates included demographics and relevant survey measures [2, 3, 10, 26] accounting for heterogeneity in covariate distributions [18].

### 2.5 Ethics statement

The study protocol was approved by the Ukrainian Institute on Public Health Policy IRB#1 (#2020‐009‐02). All participants provided informed consent.

## 3 RESULTS

Among 199 participants enrolled at baseline, 156 (78.4%) were retained at both 3 and 6 months. Baseline characteristics [18] did not differ significantly between participants who were and were not retained at follow‐up. Participants were mostly male in their mid‐30s and had been injecting drugs for a mean of 16.6 years (SD = 8.6). At 3 months, 43.3% reported recent high‐risk injecting (receptive syringe sharing, using pre‐filled syringe, back‐ or front‐loading or container sharing). About a third (29.3%) reported considering themselves at risk of acquiring HIV via injection, while 5.1% perceived being at risk through sexual intercourse. One participant tested positive for HIV at 3 months and none at 6 months [18].

Figure 1 shows the cross‐tabulation of observed TFVdp/FTCtp concentrations at 3 and 6 months. At both visits, most participants had unquantifiable levels of both metabolites (65.0% at 3 months and 72.2% at 6 months) consistent with less than 1 dose/week PrEP intake during the previous 2 months. A small proportion had TFVdp/FTCtp concentrations consistent with regular dosing of ≥4 doses/week (1.3% at 3 months and 3.2% at 6 months).

**Figure 2:**
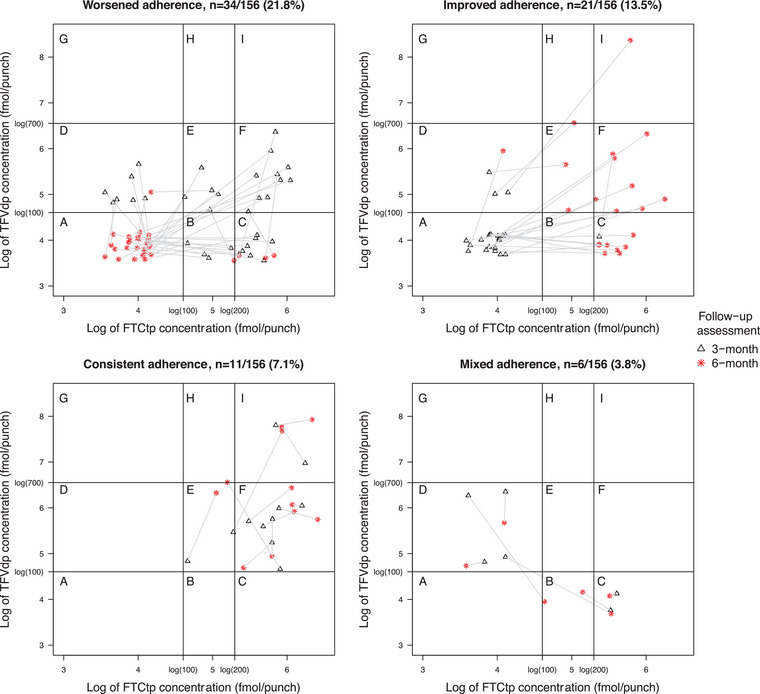
**Dynamics of TDF/FTC metabolite levels in dried blood spot between 3‐ and 6‐month assessments in the groups of participants with worsened (*n* = 34), improved (*n* = 21), consistent (*n* = 11) and mixed (*n* = 6) daily oral PrEP adherence among people who inject drugs in Kyiv, Ukraine during July 2020–March 2021. Each study participant is represented by a black triangle showing TFVdp/FTCtp concentrations at 3‐month assessment, a red star showing TFVdp/FTCtp concentrations at 6‐month assessment and a grey line connecting the two symbols to show changes in TFVdp/FTCtp concentrations for each individual. Plotting cells labelled A−I correspond to TDF/FTC metabolite concentration thresholds described in Figure** **1**. **For illustration purposes, TFVdp/FTCtp concentrations below quantification limit (100 fmol/punch for either metabolite) were imputed using random number generator (values between 35 and 65). Metabolite concentrations are plotted on a log scale**. Abbreviations: FTC, emtricitabine; FTCtp, emtricitabine triphosphate; TFV, tenofovir; TFVdp, tenofovir diphosphate.

Figure 2 illustrates the longitudinal dynamics of PrEP intake and suggests that adherence worsened over time: 21.8% demonstrated worsened and 13.5% improved adherence at 6 months compared to 3 months. While 7.1% had TFVdp/FTCtp levels consistent with regular intake of ≥2 doses/week, only 1.3% were regularly taking ≥4 doses/week. Over half (53.8%) had no detectable metabolites at both assessments. Clustering of observations in cells B/C/F suggests that “white coat compliance”—a phenomenon when patients exhibit improved adherence before the clinic visit [27]—was prevalent in our study.

The results of multinomial logistic regression (Table 1) show that both worsened and improved adherence were associated with longer drug injection duration and recent alcohol use compared to consistent non‐adherence. Worsened adherence was also associated with younger age and absence of overdose. Consistent adherence (≥2 doses/week) was associated with the study intervention (SMS reminders), younger age, employment, lower income, longer injecting drug use duration, recent high‐risk injecting, absence of overdose, perceived HIV risk through sexual intercourse and higher self‐efficacy of PrEP adherence. The analysis of correlates of improved versus worsened adherence found no associations with any of the candidate covariates (data not shown).

**Table 1:** Multinomial logistic regression: correlates of worsened, improved and consistent PrEP adherence at ≥2 doses/week compared to consistent lack of PrEP intake among PWID in Kyiv, Ukraine during July 2020–March 2021 (*N* = 150)

Characteristic	Worsened adherence (*n* = 34)			Improved adherence (*n* = 21)			Consistent adherence (*n* = 11)		
	beta	*p*‐value	aOR (95% CI)	beta	*p*‐value	aOR (95% CI)	beta	*p*‐value	aOR (95% CI)
Study arm									
no SMS	ref			ref			ref		
SMS	0.25	0.5883	1.29 (0.52–3.21)	0.06	0.9098	1.06 (0.36–3.12)	**2.47**	**0.0443**	**11.81 (1.07–>100)**
Age (years)	–**0.15**	**0.0145**	**0.86 (0.76–0.97)**	–0.03	0.6176	0.97 (0.85–1.10)	–**0.35**	**0.0059**	**0.71 (0.55–0.91)**
Sex									
female	ref			ref			ref		
male	–0.46	0.4183	0.63 (0.21–1.92)	–0.84	0.2089	0.43 (0.12–1.60)	–0.48	0.6824	0.62 (0.06–6.11)
Marital status									
partner	ref			ref			ref		
single	–0.10	0.8447	0.91 (0.34–2.40)	–0.31	0.6206	0.74 (0.22–2.48)	–1.93	0.1083	0.14 (0.01–1.53)
Employment									
employed (full or part)	ref			ref			ref		
unemployed	0.12	0.8466	1.13 (0.34–3.77)	–0.43	0.5932	0.65 (0.14–3.12)	–**4.61**	**0.0331**	**0.01 (<0.01–0.69)**
Income (UAH per month)									
≤3000	ref			ref			ref		
3001−8000	–0.79	0.2097	0.45 (0.13–1.56)	–0.92	0.2539	0.40 (0.08–1.93)	–2.42	0.0669	0.09 (0.01–1.18)
>8000	–1.16	0.0934	0.31 (0.08–1.22)	–0.82	0.3061	0.44 (0.09–2.13)	–**4.35**	**0.0037**	**0.01 (<0.01–0.24)**
Duration of injection drug use (lifetime, years)	**0.15**	**0.0070**	**1.16 (1.04–1.29)**	**0.13**	**0.0302**	**1.14 (1.01–1.28)**	**0.27**	**0.0262**	**1.30 (1.03–1.65)**
Alcohol use in the past 30 days									
no	ref			ref			ref		
yes	**1.29**	**0.0161**	**3.64 (1.27–10.41)**	**1.41**	**0.0355**	**4.10 (1.10–15.30)**	0.67	0.5560	1.96 (0.21–18.56)
High‐risk injecting in the past 30 days^a^									
no	ref			ref			ref		
yes	0.91	0.0748	2.48 (0.91–6.73)	1.02	0.0920	2.76 (0.85–9.01)	**4.43**	**0.0045**	**83.75 (3.94–>100)**
Overdose in the last 6 months									
no	ref			ref			ref		
yes	–**15.35**	**<0.0001**	**<0.01 (<0.01)**	–0.91	0.3665	0.40 (0.06–2.90)	–**26.75**	**<0.0001**	**<0.01 (<0.01)**
MOUD at present									
no	ref			ref			ref		
yes	–0.18	0.7264	0.83 (0.30–2.31)	0.50	0.3909	1.65 (0.52–5.21)	–1.27	0.2799	0.28 (0.03–2.81)
Depression (PHQ‐9)									
none or mild	ref			ref			ref		
moderate to severe	–0.11	0.8225	0.90 (0.35–2.32)	0.35	0.5431	1.42 (0.46–4.41)	1.40	0.1914	4.06 (0.50–33.24)
Perceived HIV risk through injection									
no	ref			ref			ref		
yes	0.05	0.9263	1.05 (0.37–2.94)	–0.45	0.4846	0.64 (0.18–2.23)	–0.96	0.3651	0.38 (0.05–3.06)
Perceived HIV risk through sexual intercourse									
no	ref			ref			ref		
yes	2.50	0.0936	12.16 (0.66–>100)	1.88	0.2306	6.55 (0.30–>100)	**8.76**	**0.0008**	**>100 (38.83–>100)**
Self‐efficacy of adherence to daily PrEP last month									
poor to moderate	ref			ref			ref		
good to excellent	0.43	0.6401	1.54 (0.25–9.35)	0.02	0.9809	1.02 (0.16–6.48)	**15.94**	**<0.0001**	**>100 (>100)**

*Note*: In the multinomial regression, the reference group was participants with consistent lack of PrEP adherence across 3‐ and 6‐month assessments (*N* = 84). All time‐varying covariates were measured at 3‐month assessment visit. Odds ratios lower than 0.01 are reported as “<0.01” and odds ratios higher than 100 are reported as “>100.” Associations significant at 0.05 level are bolded. There were no missing values on individual variables due to survey implementation via computer‐assisted instruments.

Abbreviations: aOR, adjusted odds ratio; CI, confidence interval; MOUD, medications for opioid use disorder; PHQ‐9, patient health questionnaire, 9‐item version; PrEP, pre‐exposure prophylaxis; PWID, people who inject drugs; SMS, short messages service; UAH, Ukrainian hryvnya (Ukraine national currency).

^a^ High‐risk injecting includes self‐report of any of the following: receptive syringe sharing, using pre‐filled syringe, back‐ or front‐loading or container sharing.

## 4 DISCUSSION

To the best of our knowledge, this is the first study that investigated PrEP adherence dynamics among PWID leveraging distinct pharmacokinetic profiles of two PrEP metabolites. The parent study [18] was designed to model a real‐life community‐based PrEP programme: participants received PrEP for take‐home dosing, no incentives were offered for intake, and counselling and information were provided in accordance with the national protocols; participants were not penalized for low adherence.

We found that overall PrEP compliance was low and worsened as time progressed, with only 7.1% of participants showing evidence of consistent intake of ≥2 doses/week, and 1.3% taking ≥4 doses/week. “White coat compliance” [27] observed in our study suggests that in a real‐life community‐based PrEP programme without regular clinical encounters and continued support, adherence may be even lower. This emphasizes that while PrEP offers the potential in reducing HIV transmission among PWID, it may not produce the expected effect in isolation and must be viewed as a component of a comprehensive patient‐centred harm‐reduction package designed to address individual and structural barriers [28]. PrEP should be available, accessible and offered freely to those who would like it, paired with frequent HIV testing to ensure that people who seroconvert are detected early and switched to a full antiretroviral regimen in a timely manner [29, 30]. Behavioural and pharmaceutical HIV prevention interventions with proven effectiveness, including needle/syringe programmes, medications for opioid use disorder and ongoing psychosocial support, remain critical in sustaining risk reduction in Ukraine and the eastern Europe and central Asia (EECA) region, where HIV epidemics continue to be driven by unsafe drug injection [19, 28, 31].

The parent study analysis found no significant effect of the trial intervention (SMS reminders) on PrEP adherence [18]. However, analysis of adherence patterns presented in this paper shows that consistent adherence was associated with SMS reminders compared to non‐adherence. Failure to detect the overall effect was, therefore, likely driven by the small proportion of participants who benefited from the intervention, diluting the effect size in the overall sample. In the adherent group, text messages likely served as a reminder to take a pill, while producing little to no effect among participants who may not have seen PrEP as a priority.

The parent study results suggest that self‐reported motivation and adherence may not serve as reliable predictors of PrEP intake [18]. Despite the small sample size, in the present analysis, we found many factors associated with consistent adherence that may provide practical guidance in identifying groups that are likely to benefit from PrEP and those who require additional support. One encouraging finding was that consistent PrEP adherence was associated with recent high‐risk injection and perceived HIV risk via sexual intercourse, suggesting that people at higher risk of HIV acquisition were more likely to adhere to PrEP. Association between PrEP intake and longer drug use history may further support this, as HIV may be more prevalent in networks of PWID who have used injection drugs for longer and may also have better awareness of associated risks. Perceived risk of HIV acquisition via drug injection, while more prevalent, was not associated with PrEP adherence. This may suggest that PrEP intake led to the feeling of protection and thus lower perceived risk or that high‐risk injection practices may not translate into risk perception [32], possibly due to higher risk tolerance towards more familiar practices.

We found that groups with improved and worsened adherence were similar with respect to all covariates suggesting that these people may be taking PrEP periodically rather than representing distinct subgroups who gain or lose interest in PrEP over time, but a larger sample size is needed to confirm this hypothesis. Of note, alcohol use was associated with both improved and worsened adherence, but not with consistent adherence, suggesting that alcohol use—a known risk factor for HIV acquisition [33]—may be related to unstable behaviours, in particular with respect to sexual HIV transmission [34], resulting in periodic PrEP intake.

Our study had several limitations. Despite being the largest study to date to analyse PrEP biomarkers among PWID, insufficient heterogeneity in outcomes resulted in small samples in some groups and consequently wide confidence intervals around regression coefficients. Only two participants were consistently taking ≥4 doses/week (dosing level shown to offer sufficient protection [9]), prohibiting the analysis of correlates of this outcome. While the study implementation overlapped with the COVID‐19 pandemic, evidence suggests that the impact of the pandemic on the local drug scene, harm reduction and clinical services for PWID was short‐term [35, 36] and unlikely to meaningfully affect our results.

In the past two decades, Ukraine achieved considerable progress in curbing the HIV epidemic [22, 37–39]. The ongoing war with Russia disrupted lives and put pressure on governmental and community systems that provided supporting structures to the most vulnerable [40, 41, 42, 43]. At the same time, Ukrainian society demonstrated strong resilience in the face of the crisis. Critical HIV services continue to be delivered on the territories controlled by Ukraine with a central role played by the communities of people living with HIV and people who use drugs [44, 45, 46]. Confronted with the risk of surging transmission, it is vital to leverage all available tools, including PrEP, to reduce the spread of HIV among key populations. Findings from our study suggest that in the current Ukrainian context, comprehensive integrated harm reduction strategies are more important than ever to improve the efficacy of HIV prevention through synergies.

## 5 CONCLUSIONS

While long‐acting injectable PrEP shows promise in improving adherence [30, 47–49], its efficacy has not been tested among PWID, and daily oral PrEP remains the only option currently available to this group. Without proper integration with effective risk reduction strategies and addressing structural barriers to care engagement, PrEP may be unable to achieve the effects demonstrated in clinical trials. It is especially important in the context of HIV epidemics associated with injection drug use in the EECA region and the public health crisis in Ukraine caused by the war with Russia. As adherence may worsen over time, the findings from our study offer practical guidance in identifying groups of PWID who may benefit from additional support to improve PrEP compliance. While the effect of SMS reminders was small, the intervention may be beneficial to some people. Further research involving biomarkers is needed to investigate reasons for low compliance and test interventions that may lead to better outcomes, including case management, peer navigation, cash incentives, and determining the efficacy and the implementation modalities of long‐acting injectable PrEP among PWID.

## COMPETING INTERESTS

The authors report no competing interests.

### AUTHORS’ CONTRIBUTIONS

OM: Conceptualization, methodology, formal analysis, supervision, validation, writing—original draft, writing—review and editing. MK: Conceptualization, funding acquisition, investigation, writing—review and editing. OM: Project administration, writing—review and editing. SA: Methodology, writing—review and editing. ML: Resources, writing—review and editing. OV: Funding acquisition, supervision, investigation, writing—review and editing. KD: Methodology, supervision, writing—review and editing.

## FUNDING

This research was supported by the Global Fund to Fight AIDS, Tuberculosis, and Malaria through the grant titled “Gain Momentum In Reducing TB/HIV Burden In Ukraine,” awarded to the Alliance for Public Health (Ukraine). The laboratory component was supported by the University of North Carolina at Chapel Hill Center for AIDS Research, an NIH‐funded programme P30AI050410. The funders of the study had no role in design, data collection, data analysis, data interpretation or writing of the report.

## DISCLAIMER

The content of this publication is solely the responsibility of the authors and does not necessarily represent the official views of the funders.

## Data Availability

According to the data‐sharing policy of the Alliance for Public Health, the data obtained from this study cannot be made publicly available due to privacy or ethical restrictions. However, it can be provided upon a reasonable request, which should be directed to office@aph.org.ua.
